# Methicillin-resistant *Staphylococcus aureus* containing *mecC* in Swedish dairy cows

**DOI:** 10.1186/1751-0147-55-6

**Published:** 2013-01-31

**Authors:** Helle Ericsson Unnerstad, Björn Bengtsson, Margareta Horn af Rantzien, Stefan Börjesson

**Affiliations:** 1 Department of Animal Health and Antimicrobial Strategies, National Veterinary Institute, Uppsala, SE-751 89, Sweden

**Keywords:** MRSA, *Staphylococcus aureus*, *mecC*, Mastitis, Dairy cows

## Abstract

**Background:**

Hitherto, methicillin-resistant *Staphylococcus aureus* (MRSA) has not been detected in Swedish cattle. However, due to the report of *mecC*, a novel homologue to the *mecA* gene, there was reason to re-evaluate susceptibility results from strain collections of *Staphylococcus aureus* and test suspected isolates for the presence of *mecC*.

**Findings:**

Bovine isolates of *S. aureus* with elevated minimum inhibitory concentrations of beta-lactams were retrospectively tested for presence of *mecC*. In four of the isolates *mecC* was detected.

**Conclusion:**

In Sweden, this is the first finding of MRSA in cattle and the first detection of MRSA harbouring *mecC* of domestic animal origin. MRSA in animal populations has implications as a potential reservoir with risk for spread to humans. Occurrence of MRSA among Swedish cattle appears still very limited.

## Findings

Methicillin-resistant *Staphylococcus aureus* (MRSA) first emerged as a problem in hospitals but is now also widely distributed in the community, although the prevalence varies among European countries [1]. In animal settings, MRSA has been an increasing problem for the last ten years, initially as the cause of clinical disease in companion animals and horses. It is now widely distributed in pig populations in many countries and has also been described in poultry and veal calves [2]. In most food-producing animals, MRSA rarely causes clinical disease, although there are reports of arthritis, dermatitis and sepsis in pigs [3]. In dairy cows, *S. aureus* is a common bacterial cause of mastitis and MRSA is known to cause mastitis as well [4]. Among food-producing animals, the MRSA lineage clonal complex (CC) 398 is clearly dominating. A reservoir in food-producing animals may constitute a risk for human health and can give a significant contribution to the burden of MRSA in human healthcare, at least in low prevalence countries [5]. In 2011 MRSA with a divergent *mecA* homologue, named *mecC* (formerly *mecA*_LGA251_), was detected in milk samples from dairy cows in the UK and human clinical samples in the UK, Denmark and Ireland [6,7]. The *mecC* has only ~70% similarity with *mecA* and is not identified with conventional confirmatory methods [6].

Sweden is still a country with low prevalence of MRSA infections in humans and < 1% of invasive *S. aureus* isolates are MRSA [8]. Therefore, measures to prevent a situation where animals become a reservoir for MRSA are important. The situation in production animals in Sweden is still favourable and so far MRSA has only been detected in one pig herd, indicating a low prevalence also in the pig population [9].

In order to investigate the presence of MRSA in dairy cows in Sweden, penicillinase-producing *S. aureus* isolates originating from milk samples sent to the Mastitis Laboratory, National Veterinary Institute, Uppsala, Sweden for routine bacteriology were screened for MRSA phenotype in 2010–2011. For practical reasons penicillinase-negative isolates were not tested, since it was expected that the majority of MRSA-isolates produce penicillinase.

During the time period, 8757 submissions of milk samples from cows were received. Each submission constituted a variable number of milk samples from one farm, and samples from the same farm could have been submitted more than once. In 534 (6%) of the submissions, at least one sample contained *S. aureus* and in 207 submissions (2%), penicillinase-producing *S. aureus* was detected in the routine testing using the “clover-leaf” method [10]. The samples emanated from dairy farms in different parts of Sweden but the MRSA-screening was performed anonymously and epidemiological information of individual samples, such as geographical origin, was not known. Samples most likely originated from cows with mastitis although clinical data on each cow were not available. During January to April 2010, all penicillinase-producing isolates were tested for resistance to oxacillin and cefoxitin by microdilution using the VetMIC MRS panel (National Veterinary Institute, Uppsala, Sweden) following CLSI standard M31-A3 [11], but during the remaining period only one isolate per submission was tested. In total, this resulted in 311 penicillinase-producing isolates analysed by microdilution and seven of these were suspected of being MRSA due to elevated minimum inhibitory concentrations (MICs) of oxacillin and cefoxitin, but the *mecA* gene was not detected with polymerase chain reaction (PCR). When knowledge about the novel *mecA* homologue became available, our results were re-evaluated and all isolates with MICs of oxacillin (with 2% NaCl) and /or cefoxitin ≥ 4 mg/L (in total 58 isolates), were tested for the presence of *mecA* and *mecC* with PCR [6]. Four of these isolates were verified as MRSA carrying *mecC*.

The confirmed MRSA isolates were further characterized by *spa*-typing [12], multilocus sequence typing (MLST) [13], pulsed-field gel electrophoresis (PFGE) [14] and detection of the Panton Valentine leukocidine (PVL) toxin genes [15]. They were also tested for antimicrobial susceptibility to cephalothin, chloramphenicol, ciprofloxacin, clindamycin, erythromycin, fucidic acid, gentamicin, kanamycin, oxacillin, penicillin, tetracycline and trimethoprim by microdilution using the VetMIC GP-mo panel (National Veterinary Institute, Uppsala, Sweden) following CLSI standard M31-A3 [11] Three of the isolates, originating from milk samples collected in 2010, were of *spa*-type t524 (*spa* repeat succession 04–17) and ST130 (CC130) and had identical PFGE-patterns. One isolate, originating from a milk sample collected in 2011, was of *spa*-type t9111 (14-44-12-23-18-17-17-17-23-24), ST425 (CC425) and showed a PFGE-pattern different from the other three isolates. The PVL-genes were not detected in any of the isolates. All four isolates were resistant to beta-lactams, but susceptible to all other antimicrobials tested. The isolates were confirmed to produce penicillinase which is noteworthy because, to our knowledge, penicillinase production has not been reported in isolates with *mecC* despite presence of the beta-lactamase gene *blaZ*[6].

As part of the surveillance of antimicrobial resistance in mastitis pathogens from dairy cows in Sweden, several studies have been performed during the last ten years without findings of MRSA. Isolates in these studies have been tested for antimicrobial susceptibility as described above. Results of susceptibility testing of 419 isolates of *S. aureus* from dairy cows since 2001 were available for re-evaluation. In this material, 39 isolates were subjected to testing for the presence of *mecA* and *mecC* with PCR, but MRSA was not detected.

In total, 730 (311 + 419) isolates were available for investigation, but only four were confirmed to be MRSA indicating a low prevalence among dairy cows in Sweden, at least in association with mastitis. MRSA with *mecC* has also been identified in human clinical samples in Sweden [16]. Since the geographic origin of the bovine isolates is unknown it is not possible to investigate epidemiological links between isolates or links to human isolates of MRSA from Swedish healthcare. The Swedish bovine isolates belonged to the same MLST:s identified in bovine milk samples in the UK and in human samples from the UK, Denmark, Ireland and Germany [6,7,17]. However, none of the Swedish bovine isolates was of *spa*-type t843 (04-82-17-25-17-25-25-16-17), which appears to be common in other countries [6,17,18]. It has been suggested that MRSA CC130 might be bovine associated, but further studies are needed to establish if this truly is the case [6].

In conclusion, this is the first finding of MRSA in Swedish cattle, as well as the first detection of MRSA with *mecC* of domestic animal origin in Sweden. In addition, occurrence of MRSA among Swedish cattle appears still very limited.

## Competing interests

The authors declare that they have no competing interests.

## Authors’ contributions

HEU participated in the design of the study and drafted the manuscript. BB participated in the design of the study and helped to draft the manuscript. MHR participated in the design of the study and carried out the susceptibility testing. SB participated in the design of the study, helped to draft the manuscript and carried out the molecular analyses. All authors read and approved the final manuscript.
